# Recurrent Miller Fisher Syndrome: A Case Report

**DOI:** 10.5811/cpcem.47931

**Published:** 2026-01-20

**Authors:** Larry Vernier, Griffin Barnes Gilmore, Kelsey Van Housen, Xiao Chi Zhang

**Affiliations:** * Montefiore Einstein, Department of Emergency Medicine, The Bronx, New York; † Mount Sinai Elmhurst, Department of Emergency Medicine, Queens, New York; ‡ Sidney Kimmel Medical College, Thomas Jefferson University, Philadelphia, Pennsylvania; § Thomas Jefferson University, Department of Emergency Medicine, Philadelphia, Pennsylvania

**Keywords:** Guillain-Barré syndrome, Miller Fisher syndrome, neurological emergencies, case report

## Abstract

**Introduction:**

Guillain-Barré syndrome can manifest with progressive paralysis, requiring careful monitoring and treatment with steroids or intravenous (IV) immunoglobin. While this disease can be devastating and require intensive care unit level of care, there are few incidences of relapses.

**Case Report:**

A 67-year-old man with a past medical history of Miller Fisher syndrome variant of Guillain-Barré syndrome, with complete recovery, presented to the emergency department (ED) with ataxia, ophthalmoplegia, dysphonia, and ambulatory dysfunction. The patient noticed his neurologic symptoms after waking up, and he presented to the ED with physical examination positive for difficulty with upward gaze, mild right lower facial droop, dysmetria with left finger to nose testing, and unsteady gait. A lumbar puncture revealed albuminocytologic dissociation (an elevated cerebrospinal fluid protein count without elevation in white blood cell count) and he was diagnosed with recurrent Miller Fisher syndrome. The patient completed a five-day course of IV immunoglobin with marked improvement of his symptoms. Unfortunately, the patient continued to have ambulatory difficulty, requiring inpatient rehabilitation with residual deficits including ophthalmoplegia and mild dysphonia.

**Conclusion:**

Miller Fisher syndrome is an uncommon variant of Guillain-Barré syndrome with atypical neurologic findings that can lead to respiratory distress; it requires high levels of suspicion and diagnostic evaluation. This disease process can recur in a patient’s lifetime, contrary to what has been reported in the literature.

## INTRODUCTION

Miller Fisher syndrome is an uncommon variant of Guillain-Barré syndrome that can result in progressive paralysis and has been found to have relapses, contrary to common medical knowledge.^1^,^2^ Guillain-Barré syndrome is an autoimmune disease caused by an antibody attack of the myelinated sheath of peripheral nerves in the body resulting from molecular mimicry.^3^ The symptoms typically present as ascending lower motor nerve dysfunction starting in the lower extremities. Miller Fisher syndrome is a rare variant of Guillain-Barré syndrome that initially presents as cranial and facial nerve dysfunction causing symptoms of ophthalmoplegia, ataxia, and areflexia, before further progressive neurologic deterioration resulting in chest wall weakness and severe respiratory distress.^1^,^4^ While most cases resolve within 8–12 weeks without recurrence when treated appropriately with intravenous (IV) immunoglobulin,^3^ recurrence of any variant of Guillain-Barré syndrome, including Miller Fisher syndrome, is rare.

## CASE REPORT

A 67-year-old man with a past medical history of hypertension, Crohn colitis, basal cell carcinoma, and Guillain-Barré syndrome presented to the emergency department (ED) with several hours of ophthalmoplegia, speech changes, and ambulatory dysfunction. He first noticed these symptoms upon waking in the morning and promptly presented to the ED. He reported diplopia, headache, voice changes, and bilateral lower extremity paresthesias. Review of systems was notable for cough and congestion for the previous few days. He noted that these neurologic symptoms mirrored his original diagnosis of Miller Fisher syndrome five years prior, specifically the GQ1b-positive Miller Fisher variant, with complete resolution within four months after hospitalization.

Upon ED arrival, his vital signs were as follows: heart rate, 86 beats per minute; blood pressure, 155/73 millimeters of mercury; respiratory rate, 16 breaths per minute; oxygen saturation, 97% on room air; and temperature, 98.9 °Fahrenheit. On examination, he was alert and oriented times four, and his speech was regular without dysarthria or dysphagia. His cranial nerve exam revealed symmetric facies, intact hearing, and symmetric palate. His motor exam showed normal bulk and tone, 5/5 strength in all extremities. A detailed neurologic exam, however, revealed mildly diminished sensation over fingertips, left finger-to-nose dysmetria, truncal ataxia, and inability to ambulate without assistance. He was noted to have dysphonia with hypernasality and hoarseness without aphasia. Fund of knowledge was appropriate. All other exam findings were within normal limits.

Laboratory results showed a complete blood count with a white blood count of 7,000 cells/microliter (**μL**) (reference range: 4,500–11,000 cells/**μL**) and a hemoglobin of 13 grams per deciliter (g/dL) (14–18 g/dL). His metabolic panel was notable for a glucose of 135 (70–99); his chest radiograph and negative inspiratory force study were normal. A non-contrast computed tomography (CT) of the brain showed no acute intracranial hemorrhage or mass effect; however, there were findings suggesting a new but not acute left parietal white matter infarct. Negative inspiratory force was reassuring. A lumbar puncture revealed an opening pressure of 14 cm H2O (10–25); clear cerebrospinal fluid with 2 white blood cells per μL (0–5 μL); 0 red blood cells/μL (0–10 μL); glucose, 65 mg/dL (45–80 mg/dL); protein, 56 mg/dL (15–45 mg/dL); and negative cerebral spinal fluid gram stain. The patient was diagnosed with recurrent Guillain-Barré syndrome, Miller Fisher variant, and admitted to the neurology service.

During his hospitalization, the patient underwent magnetic resonance imaging (MRI) that showed no acute infarct but probable subacute to chronic infarct in the left corona radiata. Antibody studies confirmed positive Gq1b, similar to his previous antibody testing five years prior, confirming his recurrent Miller Fisher syndrome diagnosis. The patient completed a five-day course of IV immunoglobulin with marked improvement of his symptoms. Unfortunately, the patient continued to have ambulatory difficulty, requiring inpatient rehabilitation with residual deficits including ophthalmoplegia and mild dysphonia.

## DISCUSSION

Guillain-Barré syndrome and Miller Fisher syndrome are uncommon but treatable causes of progressive paralysis.^5^ Guillain-Barré syndrome is a post-infectious, immune-modulated neuropathy that can lead to symmetrical ascending paralysis. There is an incidence of 0.4 to 2 per 100,000 with two-thirds of cases caused by an antecedent infection of *campylobacter jejuni*, leading to molecular mimicry that causes the immune system to target gangliosides in the neurons and slowing neuron conduction.^6^ The disease reaches its nadir usually around four weeks and recurrence is uncommon, estimated to happen in < 10% of all cases.^7^

*CPC-EM Capsule*

What do we already know about this clinical entity?

*Miller-Fisher syndrome (MFS) is an uncommon variant of Guillain-Barré Syndrome*

*with ophthalmoplegia, ataxia, and areflexia.*

What makes this presentation of disease reportable?

*Miller-Fisher syndrome recurrences are extremely rare, and our patient presented with a recurrence of MFS with similar symptoms after completing initial treatment.*

What is the major learning point?

*Miller Fisher Syndrome can recur in a patient’s lifetime.*

How might this improve emergency medicine practice?

*Miller Fisher Syndrome can lead to respiratory distress that requires high levels of suspicion and diagnostic evaluation.*

Miller Fisher syndrome is a variant of Guillain-Barré syndrome characterized by at least two of the following: ataxia, areflexia, and ophthalmoplegia^3^; it is believed to be responsible for 5–25% of cases of Guillain-Barré syndrome.^6^ Miller Fisher syndrome is thought to be caused by molecular mimicry due to a preceding infection (approximately two-thirds of case presentations), reaching a nadir around four weeks, similar to Guillain-Barré. While Guillain-Barré syndrome targets primarily peripheral nerves, resulting in progressive paralysis, Miller Fisher syndrome causes demyelination in cranial nerves as well, generally cranial nerves III, IV, and VI, resulting in ophthalmoplegia and diplopia. In most cases, Miller Fisher syndrome does not recur; however, variants have been observed that recur many times over several years.^8^

It is common for Guillain-Barré syndrome and Miller Fisher syndrome to overlap in presentation.^6^ A textbook presentation of either disease is very unlikely. For example, some people with typical Guillain-Barré syndrome will have ophthalmoplegia, and patients with Miller Fisher syndrome sometimes report ascending numbness.

Fortunately, Guillain-Barré syndrome and Miller Fisher syndrome require similar diagnostic workups and treatment. Advanced brain images such as CT or MRI may be obtained but are not sufficient to diagnose either of these etiologies of progressive neurological disease.^6^ The gold standard is to obtain cerebrospinal fluid CSF analysis; a positive result would demonstrate increased protein levels without an increase in mononuclear or polynuclear cells, a finding described as albuminocytologic dissociation.^5^,^6^ Although this finding is most indicative of Guillain-Barré syndrome and its subtypes, if the clinical history is consistent enough it is still possible to diagnose Guillain-Barré syndrome and its variants without this finding.

Guillain-Barré syndrome and Miller Fishe syndrome are not the only causes of ascending paralysis. Diseases such as tick-borne paralysis and electrolyte abnormalities have similar presentations, including hypomagnesemia and hypokalemia, and should be considered on the differential diagnosis for ascending paralysis.^9^ Wernicke encephalopathy, characterized by nystagmus, ataxia, and confusion, has been shown to be commonly confused as Guillain-Barré syndrome.^9^ Other infectious causes of peripheral polyneuropathy such as HIV could be confounded with Guillain-Barré. Finally, neuromuscular junction diseases such as myasthenia gravis and Lambert-Eaton syndrome that present with persistent weakness have been known to be confused as Guillain-Barré or Miller Fisher syndrome.^9^

The treatment primarily consists of IV immunoglobulin or plasma exchange with respiratory rescue as indicated. Diagnostically, antibody tests for precedent infections can be useful in diagnosing Guillain-Barré syndrome; specifically, anti-GQ1b antibodies are present in 90% of Miller Fisher syndrome cases. Electrodiagnostic studies can also be helpful in diagnosis.^6^

Patients at risk of imminent respiratory collapse or severe cardiovascular dysfunction will require intensive care unit (ICU) admission.^6^ Criteria requiring ICU admission include rapid progression of respiratory muscle weakness, evolving respiratory distress, and severe dysautonomia and dysphagia.^10^ Up to 22% of patients admitted for Guillain-Barré syndrome require mechanical ventilation; therefore, there should be a low threshold for admitting patients to the ICU who appear to be at risk of respiratory distress or cardiovascular collapse. Rapid decline of the expiratory forced vital capacities to < 15 mL/kilogram of ideal body weight, or of the negative inspiratory force to < 60 cm/H2O, each indicate the need for urgent intubation and mechanical ventilation.^11^

Albuminocytologic dissociation is usually diagnostic for both diseases; however, patients with concerning clinical history for Guillain-Barré syndrome and its variants should be worked up despite a normal cerebrospinal fluid analysis. These patients require neurological consultation and possible admission for IV immunoglobulin or plasma exchange. There should be a low threshold for ICU evaluation and admission in patients diagnosed with these diseases.

Furthermore, although Guillain-Barré syndrome and Miller Fisher syndrome are established to have a low chance of recurrence, it is not impossible to have multiple courses of these diseases. A history of Miller Fisher syndrome should not necessarily preclude a recurrence.^3^,^7^

## CONCLUSION

Guillain-Barré syndrome and its variant, Miller Fisher syndrome, are uncommon causes of progressive paralysis that emergency physicians should be aware of. It is important to keep in mind that they can recur in a patient’s lifetime, as seen in the presented case of Miller Fisher syndrome. Work-up with CT can be non-diagnostic, and a lumbar puncture should be performed. Patients diagnosed with Guillain-Barré syndrome require admission, respiratory evaluation, and ICU admission if there are signs of respiratory distress, severe dysautonomia or dysphagia, or imminent cardiovascular collapse.
